# Environmental DNA as Novel Technology: Lessons in Agenda Setting and Framing in News Media

**DOI:** 10.3390/ani11102874

**Published:** 2021-09-30

**Authors:** Amy Fitzgerald, Jennifer Halliday, Daniel Heath

**Affiliations:** 1 Department of Sociology, Anthropology and Criminology, University of Windsor, Windsor, ON N9B 3P4, Canada; hallidaj@uwindsor.ca; 2 The Great Lakes Institute for Environmental Research, University of Windsor, Windsor, ON N9B 3P4, Canada; dheath@uwindsor.ca; 3 Department of Integrative Biology, University of Windsor, Windsor, ON N9B 3P4, Canada

**Keywords:** environmental DNA, novel technology, environmental ethics, animal ethics, news media, framing, agenda setting

## Abstract

**Simple Summary:**

Increasing threats to wildlife have made assessing their populations, health, and adaptation to stressors ever more important. The use of environmental DNA to make these assessments is relatively new and offers many advantages, such as non-lethal sampling. How novel technologies such as these are framed in the news media is critically important because the general public gathers much of its information about scientific developments from the media, and public perceptions can impact use of technology, responses to data derived from its use, and ethical concerns. To date, media constructions of eDNA and perceptions among the general public have not been examined. The current paper begins to address this gap by undertaking an examination of media coverage of eDNA in Canada and the United States for the past approximately twenty years—likely a critical period in shaping understandings of and responses to eDNA. The findings indicate that eDNA is framed as a powerful tool, yet the social concerns that receive the most attention are those where there are financial interests at play, and these interests have to date eclipsed attending to relevant ethical considerations.

**Abstract:**

Environmental DNA (eDNA) is an emerging technology used for understanding ecosystems, environmental change, and stressors. Cellular and extracellular DNA are collected from environmental samples instead of individual wildlife animals, and as such eDNA comes with associated logistical and ethical benefits. It is increasingly being used, yet to date public knowledge and perceptions of eDNA have not been explored. Given that most of the public gathers scientific information from news media sources, this is a logical first place to start. This paper reports on a framing and agenda-setting analysis of news media coverage of eDNA in Canada and the United States from 2000 to 2020. The findings indicate that eDNA is being framed as an emerging and powerful tool, although questions regarding its validity and reliability are raised vis-à-vis identifying the presence of invasive species. Less than half of the news articles analyzed address broader social or ethical issues in relation to eDNA, and the majority focus on the potential financial impacts of eDNA findings on development projects and business interests. The potential ethical advantages of non-lethal sampling methods used via eDNA sampling are not addressed, nor are the potential ethical issues raised by its potential use in bioprospecting, indicating that the current state of agenda setting regarding eDNA in these newspapers is focused on economic impacts, to the exclusion of potential ethical issues. This unfolding news coverage will likely be key to understanding public perceptions of this novel technology.

## 1. Introduction

As the impacts of anthropogenic climate change and sustainability challenges continue to mount, there is a growing need to monitor environmental changes, assess the stressors wildlife are exposed to, and to take decisive action to mitigate—or ideally halt—the sources of these impacts. Both the natural and social sciences are needed in these efforts, yet inter-/transdisciplinary work that engages both poses challenges and remains relatively uncommon [1]. Scientific advancements are critical, but as demonstrated by the development of vaccines amidst the COVID-19 pandemic, scientific developments can only take us so far; the social sciences are needed to understand social receptivity and decision making, particularly around emerging scientific developments.

Environmental DNA (eDNA) is an emerging scientific method that can be used to better understand ecosystems, environmental change, and stressors. It could prove to be a critical tool for certain purposes/environments, such as assessing the sustainability of fish populations in freshwater bodies. The term eDNA was first referenced in 1987 as a method for extracting microbial DNA from sediments, but in the 21st century it has been extended to include small invertebrates and macroorganisms from aquatic and terrestrial sources [2,3,4]. The eDNA method entails collecting cellular and extracellular DNA from cells or organisms, both living and dead, from environmental samples—such as soil, water, or air—without the need to capture or isolate a target organism [2]. Typically, the DNA found in environmental samples is derived from sources such as decomposing organisms; shed epidermal cells from hair, skin, or scales; and bodily secretions such as feces, urine, or gametes [4,5].

Environmental DNA is a useful tool for many reasons, chief among them is that it is a relatively non-invasive way of identifying the presence or absence of organisms (e.g., endangered species, invasive species), the health of species, and food-webs. Moreover, because the samples are taken from the environment it does not require capturing or killing organisms, which has logistical and ethical advantages [6].

Given that it is a relatively new technology, there is no systematically collected information available about public knowledge and perceptions of eDNA: to the best of the authors’ knowledge, there are no peer-reviewed publications on the topic. Given that most of the general public acquires scientific knowledge from media sources [7,8,9], this is a logical first place to start in developing an understanding of how public perceptions of this novel technology, and how it might aid in wildlife conservation, are taking shape. The media can be critical in shaping public views, particularly vis-à-vis novel technologies “because the media operate at this interface between science and society, reporting on scientific advances and technological developments in specific ways, they are likely to play an important role in shaping public perceptions of new technologies and their value and applications” [9] (p. 488). News media have been a particularly important site for the transmission of information on environmental issues [10,11].

This paper undertakes an exploratory examination of how eDNA has been framed in the ‘newspapers of record’ in Canada and the United States: *The Globe and Mail* (*G&M*) and *The New York Times* (*NYT*). Before delving into the specifics of the present study, we provide an overview of the scientific literature on eDNA, focusing on its uses and potential, followed by a review of the literature focused on media constructions of science and technology, which provides the conceptual foundation for our analysis.

### 1.1. Environmental DNA: Uses and Potential

Environmental DNA is increasingly being used to document the presence or absence of target organisms, and is particularly useful for identifying rare, endangered, or invasive species [2,3,5,12]. It can also provide information on the presence or absence of particular diseases in a population [13] and can enrich understandings of trophic relationships (i.e., food webs) [14]. Environmental DNA can also be used to supplement paleoclimate and ancient DNA studies via extraction from fossils, lake sediments, peat, permafrost, and preserved gut content, which helps reconstruct ancient diets and paleoenvironments [14,15]. Finally, eDNA can be used to reconstruct whole genomes of microorganisms to better understand the microbial diversity in a given environment [2].

In contrast to eDNA, traditional live-capture research methods are often destructive and fatal, thus posing myriad ethical concerns. Lecq and colleagues [16] delineate several specific ethical issues of concern posed by lethal sampling in biodiversity studies, including furthering the decline of some wildlife populations, the inducement of pain in invertebrates and vertebrates, and non-targeted species are often accidentally caught and killed alongside targeted species. As such, the less destructive techniques used in eDNA research are preferable, although it should be noted that in some cases lethal methods are utilized to construct the reference samples used to make identifications. This advantage of eDNA was recently highlighted in a publication produced by Animal Ethics [6], a non-governmental organization focused on animal welfare.

Moreover, recent research indicates that the potential ethical advantages of eDNA research do not come at the expense of the quality of data acquired, and in fact the opposite may be true. Environmental DNA generates findings comparable to studies using physical specimens in terms of data capture and efficiency [13]. In some cases, it may be methodologically preferable to traditional methods. For instance, Strand et al. [13] found eDNA methods can reveal pathogens in aquatic populations weeks earlier than traditional capture methods, even at very low concentrations. In their study, eDNA provided valuable information about the biological status of target aquatic species in terms of their habitat, freedom from disease, early infection, mortality, and extinction. Environmental DNA monitoring has also been deemed less likely to spread infectious pathogens than traditional surveillance methods where dying organisms caught in cages can facilitate the spread of infectious diseases. It also has the potential to identify causative agents for species declines and can reveal emerging pathogens or invasive species that may otherwise go undetected unless specifically screened for [13,17], and it can reduce the risk of unintentional transfer of invasive species—a drawback of traditional sampling methods [5]. In sum, eDNA technology can facilitate higher quality, faster, and cheaper data capture. It therefore creates an opportunity for advanced biodiversity monitoring, conservation, and environmental management [3,5].

Notwithstanding these advantages, eDNA methods are not without associated challenges. Environmental DNA analyses are subject to the same issues traditional DNA analyses face, particularly those of degradation and contamination. Environmental DNA is highly susceptible to environmental conditions; therefore, the duration of preservation varies. While eDNA can preserve for up to hundreds of thousands of years in cold, dry permafrost, it can degrade in a matter of weeks in warm, moist environments [4]. Environmental DNA can also be contaminated in time and space and can reveal both current and past diversity often without a concrete way of discerning between the two [5]. Freshwater ecosystems are particularly affected by anthropogenic activity [12] and bioturbation and contact transfer could move infective pathogens upstream or downstream or contribute to a hostile ecosystem wherein the DNA degrades at a faster rate [13]. Anthropogenic and natural factors that cause DNA to move in space and time can also contribute to false positives or false negatives when looking for the presence or absence of particular species [5,13,14].

Species databases are also needed to identify specific species [16]. For example, while eDNA can be used to identify diet, it cannot distinguish between species if there is no reference DNA available [14]. The necessity of a database of species genomes introduces a new set of ethical considerations in addition to the need for lethal samples, noted above. With genetic resources, it is not the material that is being used, but rather its information and this can lead to issues of property rights, consent, and access [18]. With the formation of the International Board for Plant Genetic Diversity in 1974, genetic resources began to be thought of as common heritage. This ultimately led to the emergence of biopiracy wherein unauthorized companies in the global North accessed genetic resources and traditional knowledge from the global South for private profit, and, ultimately, these concerns caused genetic resources to become the responsibility of the state [18]. Yet, there are different regulatory frameworks that apply to human, animal, or environmental genetic information [19].

While it is primarily used for the study of non-human animals, there have been studies that have analyzed eDNA in human environments [20,21,22]. Thus, questions such as who owns DNA, what is the role and interest of business, and who is involved in the dialogue, are quite relevant [23]. Arts et al. [24] note that there is little to no international regulation and with the scientific community’s push towards ‘big data,’ questions arise as to who will fund data collection and maintenance, who should be allowed access, and who should be held accountable in the event of data control failures. Lajaunie and Wai-Loon Ho [19] also note that in addition to challenges posed by human consent for use of their DNA, there are also questions regarding which institutions might have the authority to provide consent for research involving wildlife.

Another concern regarding emerging genetic technologies is that projects can sometimes address a specific issue while simultaneously diverting or creating problems elsewhere. Digital tools that are designed for conservation purposes can sometimes be used destructively, akin to the problem of geo-tagging tourist photos inadvertently aiding efforts to poach wildlife [25]. It is therefore advisable to look beyond scientific consensus and ask what else is of concern, what else may be affected, and explore the perspectives of different stakeholders [26].

Nature conservation has a divisive history vis-à-vis social impacts, such as the displacement of Indigenous peoples and a lack of stakeholder involvement in decision making [24]. Mobilizing stakeholder involvement is preferable; however, when it comes to novel technologies, there are challenges posed by information communication to the general public.

### 1.2. Media Constructions of Science and Technology

Mass media is the central forum for debates between science and society, for it is generally through media that the general public first becomes aware of new scientific and technological advances and any associated social or ethical issues [7,8,9]. While media can be important facilitators of public education and engagement, media accounts are not neutral [8]. Media inform and provide a forum for discussion, but also selectively choose topics to cover based on public interest, and shape public perceptions through the way information is presented [11]. In short, media play an unparalleled role in not only reflecting but also shaping public perceptions and values [7,9,27].

Two conceptual tools have proven particularly useful in analyzing news media constructions of public interest issues: agenda setting and framing. Kamenova and colleagues’ [28] study of media depictions of emergent health-related technology illustrates the utility of focusing on the two interrelated processes in generating an analytical framework for understanding the impact of media on public discourse. Agenda setting is the process by which certain issues are highlighted and others excluded in an (implicit/explicit) effort to promote a certain mode of thought. In this way, the media have a significant role in setting the public policy agenda because they can make certain issues salient while marginalizing others [11,28]. Of note, ethical concerns are infrequently explicitly addressed in news stories [28]. Given that news media are the primary source of scientific information for the general public, a lapse in coverage of ethical issues can have a significant ripple effect.

Framing goes beyond what is discussed and engages with how it is discussed. Framing can involve using visuals and language as tools to stimulate the public’s alignment to a specific perspective [28]. Language is particularly powerful in framing how an issue is perceived [9]. In terms of science and technology, framing can delegitimize an otherwise valuable technology, stimulate public debate that helps reveal opportunities for improvement or advancement, or influence policy making [28], among other things.

For example, Songsore and Buzzelli [27] examined media coverage to understand how public discourse drives perceptions of wind energy development in Ontario, Canada. They argue that political agenda setting and framing determine how persuasive a message is in the media and this determines the extent to which media shapes public opinion. Similarly, Maddison and Watts [29] note how framing is used by policymakers as a tool to convert a broad social issue into a narrow policy problem that diminishes the need for broader policy change. However, framing can also influence and mobilize public interest groups [29]. Thus, media discourse helps define social problems and interested stakeholders [30]. Media analyses provide insight into these processes and are particularly useful in exposing the underlying structures of debates over technological advancements that can impact social acceptance and resistance [31].

In sum, analyses of framing and agenda setting have proven useful in examinations of media depictions of scientific and technological developments in general, and those related to the environment more specifically. We therefore utilize that approach here, drawing on early methodological conceptualization of framing analysis by Pan and Kosicki [32]. Their constructivist approach prompts analysts to take into consideration three groups of actors (journalists, their sources, and media consumers) and highlights the importance of themes in news media narratives. They define a theme as “an idea that connects different semantic elements of a story (e.g., descriptions of an action or an actor, quotes of sources, and background information) into a coherent whole … Because of this structuring function, a theme is also called a frame” (p. 59). Themes have the power to become frames because they are recognizable and thus can be experienced, can be conceptualized into concrete elements of a discourse, can be arranged or manipulated by newsmakers, and can be communicated in the ‘transportation’ sense of communications. In essence, they are tools for newsmakers to use in composing or constructing news discourse as well as psychological stimuli for audiences to process. They make a frame communicable through the news media (p. 59). This type of analysis is facilitated by examination of constituent elements, such as syntactical structures (e.g., headlines) and rhetorical structures (e.g., metaphors).

The current study examines agenda setting and framing regarding eDNA by news media in Canada and the United States, two jurisdictions where there are strong eDNA research communities and projects underway. We sought to examine the extent of coverage and temporal trends (i.e., agenda setting), the framing of eDNA across the two newspapers and across the period under examination, the extent to which the broader social/ethical implications are addressed, and finally, to assess differences between media coverage in Canada and the US. To the best of the authors’ knowledge, this constitutes the first examination of media depictions of eDNA and the first attempt to begin to understand social engagement with this new technology more generally.

## 2. Materials and Methods

We analyzed news coverage of environmental DNA in Canada and the United States published in *the Globe and Mail* and *the New York Times*, which are considered ‘newspapers of record’ in each respective country, although the *G&M* is based in Toronto and the *NYT* in New York. All stories, op-ed columns, and editorials published between January 2000 and July 2020 were analyzed. While eDNA emerged as a tool as early in the 1980s, we selected the date range from 2000 to 2020 because eDNA was not used extensively in genomics until the early 2000s. We compiled the data by searching the two newspapers using the Factiva database with the keywords “environmental DNA” and “metagenom*”. Using this method, we identified 92 articles that included these keywords.

A cursory review was conducted to determine which of these articles were indeed discussing eDNA instead of discussing other iterations of these keywords (e.g., the discussion of *DNA* in a courtroom *environment*). We determined that 42 of these items addressed eDNA: 11 in *the Globe and Mail* and 31 in *the New York Times*. This number is in line with what we expected given that eDNA is a novel technology.

We engaged in in-depth coding of these 42 articles/editorials. Our first round of coding was an inductive process that elicited coding categories. Our second round of coding entailed coding each unit of analysis (i.e., article, op-ed) using our codes. Our final round involved teasing apart some coding categories and collapsing others. We also engaged in temporal and comparative analyses to assess trends in the coding categories over time from 2000 to 2020 and between the two countries under examination here.

## 3. Results

### 3.1. Degree of Coverage

**Figure 1:**
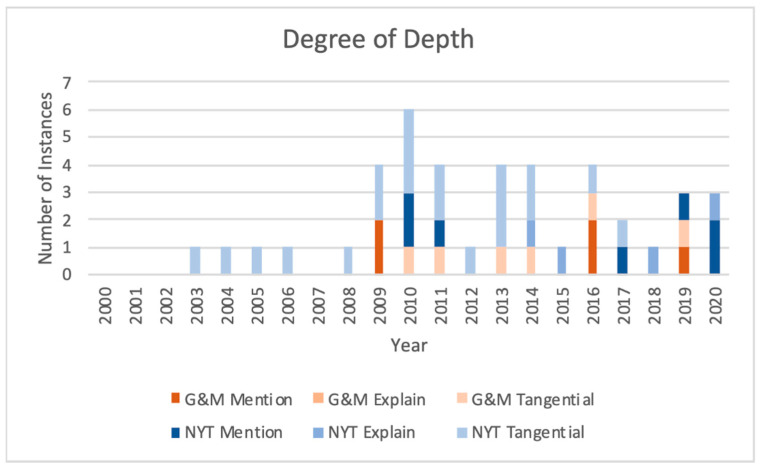
Depth of coverage of eDNA in the *Globe and Mail* and the *New York Times*, 2000–2020.

We divided the coverage into three categories to assess depth of coverage: tangential, mentioned, and explained. Twenty-six of the 42 pieces (62%) were coded as tangential; that is, eDNA was not the focus of the piece and was only mentioned tangentially in discussing the main focus of the article. For instance, in an article on Asian carp as an invasive species, the authors write “Despite electronic barriers and other efforts to contain the carp, a recent University of Notre Dame study found traces of carp DNA in Calumet Harbor, near Navy Pier and at the Wilmette pumping station” [33]. The proportion of tangential references per total newspaper coverage was approximately 55% in the *G&M* and 65% in the *NYT*. As illustrated in Figure 1, no articles meeting the inclusion criteria for analysis were published in the years 2000–2002 and 2007, those that were published prior to 2009 were merely tangential, and the proportion of tangential references has decreased over time relative to the more mentions and explains categories.

Mentions of eDNA were less common overall than tangential references, with 12 instances in total (or 29% of the total) coded as falling into this category: seven were published in the *NYT* (constituting 23% of total *NYT* coverage) and five from the *G&M* (or 45% of its coverage). Mentions became more common in both newspapers over the time period examined here. An illustrative example of the mention category can be found in an article from the *NYT* regarding a new diving site in the Caribbean, wherein the author mentions that scientists “will use an emerging technology called environmental DNA” [34] to identify species in the vicinity.

Finally, pieces that fall into the explains category were more difficult to come by. Indeed, there were only four instances in the *NYT* (approximately 10% of the total in the newspaper) and no instances were found in the *G&M*. All four of these instances appeared in the year 2014 and later. Two of these instances appeared in articles about tracking rare or endangered organisms, another on tracking invasive species, and one investigating long-extinct species. Each of these articles provide explanations for the reader of what eDNA is and provide at least some insight into what it is capable of. A recent article that appeared in the Science section of the *NYT* provides the following explanation: “Instead of digging, splashing and scraping to quantify a species’ survival, ecologists can now sample air, water, soil and even the built environment—anywhere a living creature might scrawl its genetic signature with secretions, skin or other scraps of DNA” [35].

### 3.2. Organisms of Concern

**Figure 2:**
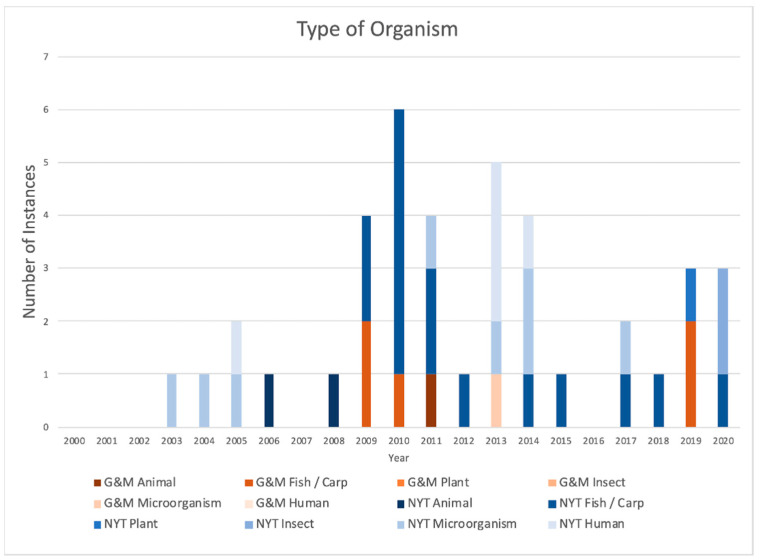
Type of organism referenced in conjunction with eDNA in the *G&M* and *NYT*, 2000–2020.

In total, 33 organisms of interest were referenced in the *NYT* and seven in the *G&M*, for a total of 40. The same trends were apparent across the newspapers as far as prevalence. Insects and plants were only infrequently mentioned (twice and once, respectively). Mammals were referenced more frequently, eight times across both newspapers, with human animals referenced five of those times. Microorganisms actually received more attention than those other categories, with nine references total. By far, however, the most commonly discussed organism of concern was fish, constituting 50% (or 20 of the total of 40) of references to specific organisms, and just over half of these were regarding Asian carp in particular. As illustrated in Figure 2, early references were focused on mammals, but beginning in 2009, fish/carp became the primary focus.

### 3.3. Framing the Usefulness of eDNA

As discussed in the literature review above, many uses of eDNA have been identified in the academic literature. We identified four main categories of uses discussed in the newspapers analyzed here: population estimation and conservation, tracking invasive species, metagenomics, and historical environmental reconstruction. It should be noted that these are not necessarily mutually exclusive categories. Therefore, we used more than one code for articles that addressed multiple uses of eDNA. There were 59 instances coded in total.

**Figure 3:**
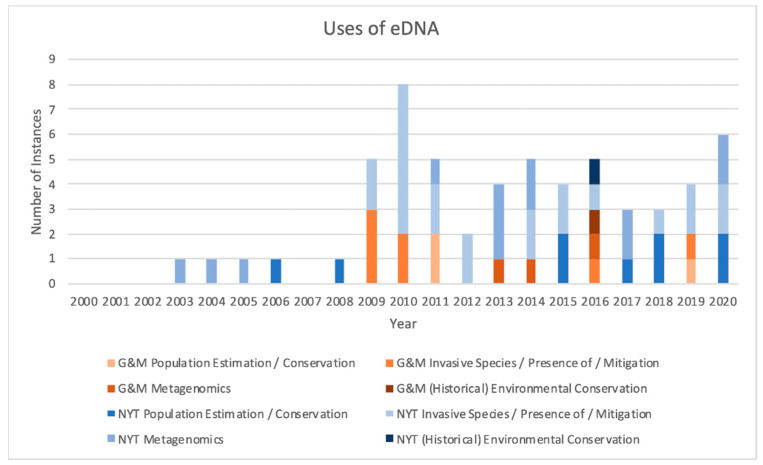
Uses of eDNA reported in the *G&M* and *NYT*, 2000–2020.

The least common use discussed in the newspapers was historical environmental reconstruction, with only two instances found: one in each of the newspapers (constituting 7% of uses covered by the *G&M* and 2% for the *NYT*). Both articles (both published in 2016) describe the use of eDNA in mapping what specific ecosystems likely looked like thousands of years ago. (See Figure 3 for an illustration of shifts over time).

Population estimation and conservation is the second most common use of eDNA discussed in the newspapers. Twelve instances fell into this category: three from the *G&M* (21%) and nine from the *NYT* (20%). On average, these appeared in the year 2014 in the *G&M* and 2015 in the *NYT*. Approximately half of the articles describe eDNA as useful in examining water bodies to assess what organisms are present and whether specific organisms of concern are present (e.g., river otters, sharks). The others described the use of eDNA to identify land and air dwelling organisms that had previously been considered extinct (e.g., stonefly) or monitoring the numbers of endangered species (e.g., Javan rhinoceros).

Environmental DNA is framed in these articles as a valuable tool for monitoring populations and of use in conservation efforts by providing insight into species declines and providing more accurate population estimates. One article describes a research project using eDNA that determined that the population of one species of concern was actually only approximately half of previous estimates [36]. According to another article, “Environmental DNA can provide some important clues about species in decline … It is hard for scientists to decide when to declare a species officially extinct, since a few stragglers may still survive unseen. But even these hard-to-find animals will still shed DNA into their environment, a signal to scientists that the species survives, if barely” [37].

The third most populous use category referenced in the newspapers is the context of metagenomics. There were 16 instances that fell into this category: 3 from the *G&M* (21%) and 13 (29%) from the *NYT*. The average years of publication of articles in this category are 2014 and 2013, respectively. The majority of the articles referred to taking eDNA samples to facilitate metagenetic analyses on bodies of water (six instances). Three instances were general descriptions of eDNA and metagenomics. The remainder were individual instances referring to metagenetic analyses of specific spaces (e.g., homes, public transit).

An early (2003) article that appeared in the Science section of the *NYT* describes this new technology as follows: “Determining the complete DNA sequence of a single species has become almost commonplace. It has been done for humans, mice, rice plants and a host of microbes, among others. Now some scientists are moving to a more audacious challenge, sequencing ‘metagenomes’, the DNA of entire ecosystems” [38] (emphasis added). The same article also references the potential metagenomics/eDNA has for identifying “thousands of previously unknown micro-organisms … as well as new drugs, chemicals and ways of harnessing bacteria to fight pollution” [38].

An article published by the same author years later in the Business section of the newspaper describes the financial and human resource costs of genome sequencing and the immense amount of data generated, and explains, “if the problem is tough for human genomes, it is far worse for the field known as metagenomics” [39]. An article appearing the following year in the Business section of the *G&M* describes growing private sector interest in genomic technologies and Canadian government investment. The potential of metagenomics is highlighted as follows: “Enter metagenomics. It’s a method for capturing a genetic snapshot of an entire community of organisms based on a mass reading of all the fragments of DNA that turn up in a particular sample. While the process has been in use for a decade to sample the diversity of microscopic life in different settings, it has only recently become inexpensive enough for many industrial applications” [40].

Specific uses were highlighted favorably. One article described the value of eDNA and metagenomics by pointing to an analysis of streams that “identified more than twice the number of organisms than traditional surveys did” [35]. Metagenomics and eDNA are framed as particularly useful vis-à-vis human health, with specific references given to assessing bacteria and viruses in human sewage [41], in homes [42], in public transit “to find out exactly what we’re breathing down there—and if any of its invisible critters are cause for worry” [43], and seawater, where “by examining the [DNA] sequences, they identified 18 kinds of disease-causing bacteria” [44]. In addition to identifying and monitoring human health risks, eDNA and metagenomics are described as also being useful for identifying “thousands of previously unknown micro-organisms … as well as new drugs, chemicals and ways of harnessing bacteria to fight pollution” [38].

Finally, and by far, the most common use of eDNA referenced in the newspaper is in relation to invasive species. Twenty-nine total instances fell into this category, accounting for 50% of the use codes in the *G&M* and approximately 49% in the *NYT*. The average years of items coded this way are 2011 and 2013, respectively. Three articles provide a general overview of the ability of eDNA to monitor for invasive species. Therein, eDNA is described as a virtual saviour in the battle against invasive species. For instance, one article, titled “Technology is invasive species’ enemy” reports “invasive species can now be detected in environmental DNA, which is found abundantly in any ecosystem. Times are changing” [45]. Another article emphasizes its utility compared to physical capture or visualization: “Now scientists only need to analyze water samples for siren eDNA: ‘You don’t have to see it to know it’s there’” [35]. Environmental DNA is therefore described as a valuable tool for knowing where to target mitigation efforts for invasive species.

Whereas one of the articles is focused on invasive plants, and another on the invasive bloody red shrimp, the majority address the threat posed to the Great Lakes by invasive Asian carp. These articles cast the carp as a financial and ecological threat, in that order. According to author, “even a whiff of them is much to worry about, especially for the Great Lakes’ $7-billion-a-year fishing industry” [46]. References to specific environmental impacts were less common, and include the following statement made in one article: “Ecologists predict that they could out-eat every other species of fish in the Great Lakes and cause the collapse of an ecosystem” [47].

Two articles explicitly articulate challenges to eDNA findings vis-à-vis Asian carp. One critique was voiced by lawyers for the Army Corps of Engineers in the U.S., quoted as challenging the eDNA finding of Asian carp in the Calumet Harbor, beyond the barriers they had constructed to keep carp out. The article explains that the state of Michigan filed a lawsuit based on the eDNA findings in an attempt to close the locks to prevent the transfer of additional carp [48]. Another article four year later states “The despised Asian carp may have finally arrived in the Great Lakes. ‘May have’ are the operative words” [49]. The article explains that one eDNA sample came back positive for Asian carp, but resampling was negative. The reporter writes “False positives can occur if a sample is contaminated, but experts largely agree that the material actually came from a silver carp” [49]. Other possibilities beyond the presence of the fish cited to explain the eDNA findings include bird droppings from those who had consumed the fish, as well as boats transferring DNA stuck to their hulls.

### 3.4. Socio-Legal and Ethical Considerations

**Figure 4:**
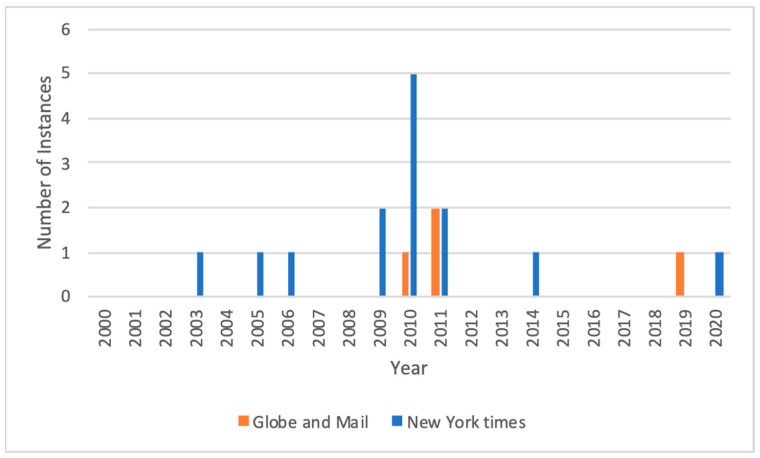
References to social and ethical considerations in conjunction with eDNA in the *G&M* and *NYT*, 2000–2020.

Overall, there were 18 articles (or approximately 43% of the total) we coded as addressing socio-political and/or ethical considerations. Four of these articles were published in the *G&M* (representing approximately 36% of articles on eDNA published in the newspaper) and 14 were published in the *NYT* (representing approximately 45% of their articles on eDNA). As illustrated in Figure 4, most of these articles are clustered in between the years of 2009 and 2011. This timing coincides with growing concern over the (potential) presence of Asian carp in the Great Lakes.

The majority of the pieces (10 of the 18) that addressed the socio-legal and/or ethical implications of eDNA discussed it in the context of the finding of Asian carp DNA using eDNA methods in the Great Lakes. This one case elucidates several (potential) socio-legal implications of eDNA technology. As one of the articles details, the current Asian carp problem was made possible by the construction of a canal years ago to link the Great Lakes to the Mississippi River. The article quotes a researcher describing the canal as “a highway to environmental havoc” [46]. The canal is relevant to the topic of eDNA because Asian carp, which were reportedly imported from Asia a few decades ago to clean algae in ponds and fish farms, breached those barriers and migrated north using the Mississippi River and the canal towards the Great Lakes. Due to their size and consumption, they have outcompeted other species in many ecosystems, and the articles point to significant concern regarding what will happen if they take hold in the Great Lakes.

The power of eDNA findings is illustrated via the Asian carp. As one article explains, “days after scientists found Asian carp DNA in North Dakota and in the Mississippi River near Minneapolis last month, Michigan, Minnesota, Ohio, Pennsylvania and Wisconsin petitioned the Supreme Court to force the corps [Army Corps of Engineers] to speed its study” [50]. The finding of Asian carp DNA ignited not only legal proceedings, but also significant social and political debate over the proper course of action, even questioning the trustworthiness of the researcher who made the discovery.

The articles describe various perspectives on how the risk posed by Asian carp should be addressed. Those interested in protecting the Great Lakes to the greatest extent possible advocated for cutting off the canal from the Great Lakes—essentially a return to the way it used to be. The next less stringent method proposed was to (temporarily) close the locks, although this would not completely keep organisms out. The importance of the Asian carp case is illustrated by the fact that the editorial board of the *NYT* published an editorial on it, in which they support the latter option. It states in part,

The only sure way to stop carp*—*and whatever other invasive species are waiting*—*is to close the canal and again separate the Mississippi and Great Lakes watersheds. That would be hugely costly and politically difficult, given the importance of shipping to the region. Closing the canal locks temporarily, while expensive and disruptive, is probably the best way to buy time until a solution can be devised that does not place an immense, fragile ecosystem entirely at the mercy of waterborne shipping. There is not a lot of time left to act.

[51]

The least stringent measure for controlling the flow of Asian carp from the river into the Great Lakes addressed in the newspapers was to stay with the status quo, that is, the extant barriers erected by the Army Corps of Engineers. A final strategy, described as complementary, involves widespread killing of Asian carps. According to one article, “anecdotal evidence from a surging carp harvest in the Illinois River seems to indicate that fishing for and selling the carp as food or processing them into fish meal or fertilizer might significantly reduce their numbers, and thus their pressure on waterways in the Chicago area” [50].

The Army Corps of Engineers and several industry groups spoke publicly in favor of the status quo and problematized the eDNA findings. The researcher who found the eDNA was referred to by spokespeople for industries that would be impacted by closing locks or otherwise blocking off the canal as “an advocate, not as a dispassionate scientist, and they vigorously dispute his recommendation that policy makers should consider ecologically separating the Mississippi River system from the Great Lakes” [52]. A lawyer representing the industry argued in court that the eDNA researcher’s views on policy should disqualify his testimony. While most of the articles about the case of Asian carp and the Great Lakes were published in the *NYT*, the one that was published in the *G&M* quoted a commentator as saying that the electronic barriers were working because only one fish’s DNA had been found on the other side of the barrier. In the article sources were also cited as referring to the lawsuits as “politically motivated” [53].

The newspaper articles make it clear that there is a lot at stake in this case, with two clear opposing sides. On the one side the Great Lakes: many of the articles cited the USD 7+ million dollar recreational fishing industry. For instance, an article describing the lawsuit describes determinations made regarding the evidence as follows: “‘Could it [Asian carp eDNA] have been from something that ate a fish?’ the judge asked about DNA found in water samples. The states’ experts believe it is more likely that the findings show the recent presence of carp. Biologists fear that if the ravenous fish get into the lakes, they could ruin the fishing industry” [54].

Industry interests, and to a lesser extent recreational interests, are positioned on the opposing side (i.e., advocating the status quo). First and foremost, the barge industry would be negatively impacted because it would no longer be able to transport materials directly between the Mississippi River and the Great Lakes. Others identified who would stand to lose include tour boat companies and inconvenienced recreational boaters. However, the possibility was also raised in one article that closing off the canal from the Great Lakes could generate jobs due to the likelihood of needing to set up new shipping and terminal facilities [33]. The Army Corps of Engineers also openly supported the status quo. A representative is quoted as saying “we need to look not only at the aquatic and ecological impact, but also the impact on the economy and the people who depend on this waterway for a living” [48]. This gives voice to a common narrative of environment versus socio-economic impacts.

In addition to the socio-economic implications of eDNA raised in the many articles focused on the case of Asian carp, a few other social issues are, to various extents, addressed in the other articles analyzed. The issues include potential impacts on development proposals; the work of managing animal populations, including identifying and dealing with competitor species; and impacts related to discoveries vis-à-vis human health.

Two *G&M* articles specifically address how findings from DNA samples from the environment can impact development proposals. One of the articles describes a group of international researchers who are using eDNA to estimate salmon populations in order to predict the well-being of whale populations, which consume salmon. It is described as “a research first that would affect everything from how whale populations are protected to the completion of Canada’s resource projects.” The article frames this eDNA research as very high stakes, describing it as possessing the “ability to safeguard an endangered whale population, [Canada’s] international standing as a steward of the marine environment, and its ability to bring to completion resource projects that impact the country’s coasts” [55].

Above and beyond impacts on development projects, eDNA is described in other articles as specifically useful for creating a baseline of wildlife populations that can be surveilled, which has become increasingly important in the larger context of global climate change. It can provide those charged with species protection with greater information, such that, for example, “marine park rangers can know what they are protecting” and more accurately track losses [56]. Or, as another author puts it, “you cannot manage what you cannot count” [55], and as such eDNA provides valuable information that facilitates the management of animal populations. It is described as particularly important for documenting the presence of endangered/nearly extinct species. As discussed in an article about the Javan rhino, DNA extracted from the environment has been useful in identifying where they are, particularly because they are so rare and therefore tracking them via other measures is challenging.

This knowledge is described as also being useful for intervening with competitor species. In the case of the Javan Rhinoceros, “the Indonesian forestry department has decided to improve rhino habitat in Ujung Kulon by keeping out or removing competitor species, like the banteng, a wild cow, and invasive, exotic plants that crowd out the rhino’s preferred food” [57]. One group of researchers are quoted as stating they are advocating for rhino habitat expansion and “hope conservation groups worldwide will help the local authorities deal with the conflicts and economic dislocation that will inevitably arise as efforts are made to expand rhino habitat in some of the most densely peopled parts of the world” [57]. Thus, eDNA findings can be used not only to impact so-called invasive species, but can also be used to impact species, including humans, that compete with valued target species.

In addition to assisting with creating baselines for species and enabling better tracking of endangered/nearly extinct species and dealing with competitor species, eDNA is framed as having some other more direct social benefits. It is described as a useful tool for detecting and fighting bioterrorism [58] and infectious diseases and antibiotic-resistant bacteria and foodborne illness [41]. It is described as having the potential to make previously unattainable discoveries that can benefit humans, as well as the environment. For instance, one article proclaims that “thousands of previously unknown micro-organisms may be unearthed, as well as new drugs, chemicals and ways of harnessing bacteria to fight pollution” [38]. Some researchers are said to also be attempting to “harness microbes to make clean-burning hydrogen fuel and reduce global warning” [38].

Only one article notes that there are corporate interests at play here, specifically in locating organisms with unique characteristics and commodifying their DNA. The author explains,

Diversa, a company in San Diego, bases its business on extracting DNA from creatures that can survive in extreme environments, such as super-hot deep-sea vents and the highly alkaline soda lakes of Kenya. It then searches the DNA for genes that provide the code for novel enzymes. One enzyme, found from sampling DNA in the soil of the tropics, is expected to cut in half the cost of a critical step in manufacturing the cholesterol-lowering drug Lipitor.

[38]

The potential ethical issues with this commodification are not explicitly addressed, and the potential implications of the social uses of eDNA are not elaborated upon beyond the cursory nods described above.

## 4. Discussion

These findings indicate that while there has not been significant attention paid to eDNA in the ‘newspapers of record’ in Canada and the U.S., interesting patterns in the coverage are evident. While all the references to eDNA found prior to 2009 were only tangential in nature, since that time, the attention paid has become more substantive. It is reasonable to expect that this trend will continue, and a high-stakes case involving eDNA—such as the case of the Asian carp and the Great Lakes—could catalyze another cluster of focused attention.

Of our three categories of degree of coverage, the most comprehensive—the explain category—was the least represented (only 10% of the total). Given the general public attains most of their information about scientific developments from the media [7,8,9], and to the extent that the findings from the news media sources analyzed here can be generalized, it would appear that as of today, the general public that relies on news media for scientific and environmental information likely has limited understanding of eDNA, its uses, and limitations.

Our comparative examination of the newspapers indicates that overall, attention to eDNA is less developed in the *G&M* than the *NYT*, both quantitatively and qualitatively. Of note, there were no detailed descriptions of eDNA in the *G&M*, only tangential references and brief mentions, whereas readers of the *NYT*, at least in most recent years, have received greater information about what eDNA is and what it is capable of. This is perhaps related to the threat posed to the Great Lakes by Asian carp, which has originated in the U.S., although it is certainly an issue that Canadians ought to be informed about as well. Moreover, the *G&M* was less likely to address socio-legal considerations in their reporting (36% of total) than the *NYT* (45%).

Our inductive coding revealed that four main uses of eDNA are addressed in the two news media sources analyzed here: historical environmental reconstruction, population estimation and conservation, metagenomics, and the detection of invasive species. In the narratives that fall into the first three categories, eDNA is framed as a powerful tool that can reach back into history and enable virtual reconstructions of ecosystems, provide greater insight into populations in decline, and even identify previously undetected species. Potential human health benefits are highlighted, such as the use of eDNA to monitor sewage, air, and water to detect potential human health threats, as well as detecting organisms that could be used to develop new drugs, chemicals, and ways to mitigate pollution.

Approximately half of the articles, however, focus on the fourth category—invasive species—and most focus specifically on Asian carp. These articles employ a narrative that frames eDNA as a powerful tool in the virtual war against Asian carp, such as referring to it as “invasive species’ enemy” [45]. Adversarial framing and war metaphors have also been observed in media depictions of diseases [59] and medications [9]. This was the only use category where critical perspectives on eDNA were included, specifically related to the case where Asian carp DNA was found on the Great Lakes side of the barrier erected to keep them out. The articles reference critiques of potential researcher bias and questioning of the validity and reliability of eDNA findings as articulated by industry interests and their legal representation, who had much to lose if waterways were blocked to mitigate the transmission of invasive species.

Overall, less than half of the articles made reference to the broader socio-legal or ethical implications of the use of eDNA, either implicitly or explicitly, and more than half of these were in reference to the case of Asian carp and the Great Lakes. Notably, although the threat posed by Asian carp is the culmination of human actions—the creation of the canal joining the Mississippi River and the Great Lakes, as well as the importation of Asian carp to clean algae in ponds and fish farms—little attention was paid to these causes ([46] is a notable exception).

The representations of the opposing positions in the Asian carp Great Lakes case are instructive in terms of agenda setting. In discussing what is at stake if Asian carp end up in the Great Lakes, most articles merely noted the USD 7+ million dollar Great Lakes recreational fishing industry. The impacts on other species or groups of people who depend on fishing for subsistence instead of recreation are not discussed, and with the exception of the piece penned by the editorial board of the *NYT,* which referenced “an immense, fragile ecosystem” [51], the ecological risks were not addressed.

Financial interests are also framed as taking front stage on the other side of the debate (i.e., among those against closing the locks or otherwise blocking the canal); the economic impacts on the barge industry and among those who would need to find alternative ways to transport materials are highlighted repeatedly. Impacts on recreational fishers and boaters were mentioned less frequently, and the potential environmental impacts of relying on other methods of transporting goods (i.e., trucking) were mentioned once. This agenda setting vis-à-vis financial interests and economic impacts over environmental impacts is consistent with other news media analyses conducted at the intersection of technology and the environment [27].

Outside of the Asian carp case, the potential socio-legal consequences of the use of eDNA are only brushed up upon. The use of eDNA to monitor for bioterrorism, viruses, and bacteria were discussed; however, the articles stop short of unpacking the implications for people who occupy regions of potential concern, such as enhanced state surveillance and how that might be experienced by already marginalized groups.

Similarly, references are made to the role eDNA can play in making determinations about wildlife populations. The implications of these findings in the form of decision-making regarding development projects and wildlife management decisions can be significant. This significance is illustrated by a statement in the *G&M* that these decisions impact Canada’s international reputation as ‘a steward of the marine environment.’ It could, therefore, have wide-ranging political economic implications. Other articles reference global climate change in particular in making the case that the population tracking made possible by eDNA is increasingly important in terms of tracking losses. It is also framed as a tool for potentially mitigating the impacts of environmental degradation through identifying bacteria and microbes that might be useful in mitigating pollution and global climate change, as well as for creating new chemicals and drugs that would benefit humans.

This utility is framed in a decidedly anthropocentric manner: while eDNA is described as enhancing our management capacities vis-à-vis other species and the environment, the causes of this assumed necessity are not explored. Perhaps this is unsurprising given that news media are limited in the amount of space that can be devoted to a specific topic. But this does not obviate the significance of what information is being transmitted to the general public: eDNA is framed as a tool for estimating the degree of environmental decline, but the environmental decline itself is not problematized. This provides a useful illustration of some of the concerns articulated by critics of the ecological modernization perspective, who accuse ecological modernizationists of privileging technological innovations over problematizing the anthropogenic origins of environmental degradation and advocating for systemic change (see, for example, [60]).

In the news articles analyzed here, there was one brief mention of corporate interest in using eDNA to locate and commodify DNA and novel enzymes, but the potential implications were not discussed. Although not explicitly identified as such by the news articles, eDNA could potentially become a valuable tool in bioprospecting. This is significant and warrants further attention as eDNA could be used in ways that end up negatively impacting the environment, and groups of non-human and human animals by extension. This potential has been acknowledged by organizations such as Genome British Columbia, which notes on its website (https://www.genomebc.ca/infobulletins/edna, accessed on 10 July 2021) that “mineral and hydrocarbon detection or ‘bioprospecting’ also has potential through the application of eDNA. Bacteria living on mineral deposits can be used to map geological formations for minerals, metals and hydrocarbons. Bacteria which live on certain minerals and deposits can offer a ‘map’ to those deposits such as springs and oil wells.” In situations where there are private interests, it is critical that attention is paid to other stakeholders and communities that may be affected (see [61] for a discussion of this point) instead of allowing private commodification to become the only—or most important—consideration. While it is important that projects employing eDNA attend to these broader constituencies, it is also important to understand that if these potentially very important social/ethical issues are not receiving media attention, they are certainly less likely to come to the attention of the general public, and researchers are less likely to experience external pressure to attend to these myriad issues of (potential) concern.

Likewise, the analysis indicates that eDNA is framed as a powerful tool in facilitating the targeted killing of species defined as invasive and competitors, but the potential ethical implications are not addressed. In the case of Asian carp, for instance, the ethical implications of mass killing were not addressed in the news reports. Ethical considerations are also not raised in the context of using eDNA to identify species of interest and then targeting competitor species for removal—non-human animals presumably by lethal means and humans vis-à-vis ‘economic dislocation.’ The proposed ‘economic dislocation’ referenced was the one instance where the environment was framed as trumping economic interests. Notably, the requisite economic dislocation discussed was in Indonesia as a means to the end of protecting the rhinoceros population, whereas suggestions of economic disruption in North America to protect the Great Lakes were much more tempered.

It is also interesting that none of the news articles addressed the ethical advantages of the non-lethal sampling made possible via eDNA methods. As noted in the literature review, one of the key advantages of eDNA methods is they do not require catching (and injuring or killing) animals [6]. This has obvious animal welfare benefits, as well as ecological benefits in terms of not killing or risking harming members of threatened/engendered species. This omission may speak to the relative importance the news media places on animal and environmental ethics, decisions made by news media based on the presumed issue salience among the general public, and/or how the benefits of eDNA methods are being communicated by experts to the media.

Our findings regarding the dearth of attention paid to the ethical considerations among news reporting are certainly not specific to the topic of eDNA; Kamenova and colleagues [28] have observed that ethical concerns frequently go unaddressed in the news media. Yet this vacuum, in combination with our other findings regarding the framing of eDNA in the news media, is important in terms of its broader implications. Pan and Kosicku’s words in their delineation of framing analysis are instructive here: “Choices of words and their organization into news stories are not trivial matters. They hold great power in setting the context for debate, defining issues under consideration, summoning a variety of mental representations, and providing the basic tools to discuss the issues at hand” [32] (p. 70). This power is expressed not only vis-à-vis what is explicitly addressed by news media, but also through what goes unaddressed.

One clear limitation of this study is that although it provides insight into framing and agenda setting regarding emergent eDNA technology, it is unable to assess how it is received and understood by the public. Such research is needed, particularly because the use of eDNA is likely to expand and public understandings will become critical in relation to adoption of this technology and policymaking. Moreover, given the increasing reliance on social media for information, analyzing reader comments provided on news content related to eDNA posted by news outlets on social media platforms might provide particularly useful insights, for instance. In addition to focused research on public understanding and perceptions, we also recommend research on the socio-legal and ethical implications of this novel technology as it continues to unfold. Finally, we acknowledge that our analysis may be limited by the focus on content in the *G&M* and *NYT*. Accordingly, it may be productive for future research to examine news media accounts in more regional-level outlets proximate to areas of specific concern (e.g., the Great Lakes).

## 5. Conclusions

The academic literature indicates that the value of eDNA methods has become increasingly apparent, particularly over the past twenty or so years, and its use is expected to continue to expand and become ever more important in the face of global climate change and other environmental threats. It is, however, still a novel technology in the broad sense, and how it is framed in the news media is critically important because the general public receives so much of its information about scientific developments and uses from the media; as such, the current time period is likely a critical window in shaping how eDNA will be understood and received. This exploratory study provides the first examination of this unfolding process in Canada and the United States.

Our analysis concludes that while eDNA has received greater quantitative and qualitative news media attention in recent years, its utility continues to be framed largely in anthropocentric terms, as a tool for rooting out invasive and competitor species, and to identify (micro)organisms that might be commodified to enhance human health. Moreover, the potential social and ethical implications of eDNA are addressed in less than half of the articles. Notably, the benefits it presents in terms of enabling nonlethal sampling have been entirely overlooked to date, and even the potential benefits to ecosystems have taken a back seat to the potential economic implications.

As illustrated in the finding of invasive species DNA (Asian carp) via eDNA methods on the Great Lakes side of the barrier erected to keep them out, economic interests can potentially be significantly impacted by this new technology. As one news article notes, if eDNA findings are powerful enough to prompt demands to shut down waterways into the Great Lakes, it could conceivably be used to shut down the St. Lawrence Seaway if invasive species of concern are identified [48]. Although this statement is to some degree exaggerative and speculative, it has the intended function of highlighting the potential of eDNA to impact economic interests. In doing so, it also points to the potential of this novel technology to become caught up in the widespread ‘environment versus economy’ narrative. The power of eDNA and its expanding use likely mean that media coverage will intensify, and as argued herein, this unfolding coverage will likely be key to understanding public perceptions of this novel technology.

## Data Availability

Not applicable.
